# Comparison between measurable residual disease relapse and morphologic relapse in acute myeloid leukemia and high-grade myeloid neoplasms

**DOI:** 10.1038/s41375-023-01981-2

**Published:** 2023-07-31

**Authors:** Lauren Shih, Megan Othus, Kelda Schonhoff, Carole Shaw, Jacob Appelbaum, Anna B. Halpern, Pamela S. Becker, Roland B. Walter, Elihu Estey, Mary-Elizabeth Percival

**Affiliations:** 1 Department of Medicine, University of Washington, Seattle, WA USA; 2 Public Health Sciences Division, Fred Hutchinson Cancer Center, Seattle, WA USA; 3 Clinical Research Division, Fred Hutchinson Cancer Center, Seattle, WA USA; 4 Department of Hematology and Hematopoietic Cell Transplantation, City of Hope, Duarte, CA USA; 5 Department of Laboratory Medicine & Pathology, University of Washington, Seattle, WA USA; 6 Department of Epidemiology, University of Washington, Seattle, WA USA

**Keywords:** Acute myeloid leukaemia, Chemotherapy

## To the Editor:

Cure of acute myeloid leukemia (AML) is unlikely without achievement of complete remission (CR), including <5% bone marrow blasts by morphology and absence of circulating blasts or extramedullary disease, as well as peripheral count recovery with absolute neutrophil count >1000/µl and platelet count >100,000/µl [1]. However, many CRs are transient, suggesting the importance of measurable residual disease (MRD) [2–4]. MRD is associated with an increased risk of later morphologic relapse despite chemotherapy or allogeneic hematopoietic cell transplantation (HCT) [2, 5, 6]. Consequently, current guidelines recognize CR with and without MRD as distinct response categories [1]. One sensitive and quantitative method for MRD detection is multiparametric flow cytometry (MFC).

The prognosis of relapse detected first as MRD (<5% marrow blasts) as opposed to full relapse (≥5% marrow blasts) is uncertain, which makes management of MRD challenging [4]. If overall survival (OS) after MRD relapse resembled OS after morphologic relapse, strategies to eliminate MRD may be justified; however, if the prognosis of MRD relapse was considerably better than that of morphologic relapse, elimination of MRD may be pursued more cautiously. Here, we compare OS after development of relapse detected first as MRD only relapse [REL-MRD] or morphologic relapse [REL-MORPH] in patients initially achieving CR without MRD [MRDneg-CR].

We identified 432 adults who received initial treatment at the University of Washington/Fred Hutchinson Cancer Center from 2008-2017 who achieved MRDneg-CR (including absence of any abnormal blasts by MFC in the marrow). Institutional review board approval was obtained. Patients were diagnosed with AML (de novo or secondary) or a high grade myeloid neoplasm (with ≥10% blasts, most commonly myelodysplastic syndrome with excess blasts-2 (MDS EB-2) or chronic myelomonocytic leukemia-2 (CMML-2)), as these groups have similar treatment and outcomes as AML patients) [7, 8]. Initial treatment course was classified as high intensity (high dose cytarabine-containing combinations, defined as cytarabine ≥1 g/m^2^/day), low intensity (azacitidine or decitabine), or intermediate (“7 + 3” or similar). Subsequent treatments, including allogeneic HCT or additional chemotherapy regimens, were recorded.

Patients attaining MRDneg-CR were subsequently classified as either ongoing MRDneg-CR, MRD positive relapse (defined as any amount of detectable abnormal blasts <5% by MFC in peripheral blood or marrow sample; REL-MRD), or morphologic relapse (≥5% blasts by peripheral blood or marrow sample; REL-MORPH) [9]. All MFC testing was completed at our institution. The treatments for REL-MRD patients were recorded, along with response to therapy (MRDneg-CR, persistent REL-MRD status, progression to REL-MORPH, death).

Landmark OS was measured from 1 year after the date patients achieved MRDneg-CR with patients last known to be alive censored at last contact. Patients who had died or were censored before the landmark time of 1 year were excluded. Analyses of OS was stratified by relapse status (MRDneg-CR, REL-MRD, REL-MORPH) at the landmark time. Multivariable Cox regression models were used to examine covariate associations with OS. Two-sided p-values of 0.05 or less were considered statistically significant. Analyses were performed using R version 4.1.1.

A total of 288 of the 432 patients remained in MRDneg-CR at last contact, while 44 developed REL-MRD and 100 REL-MORPH. Median follow up among patients alive at last contact was 8.5 years. No significant differences among gender, initial blast percentage <20%, secondary disease status, European Leukemia Net 2017 (ELN) risk category, time to MRDneg-CR, or initial treatment intensity were identified across the three groups. Patients who developed REL-MRD were older than patients without relapse (median age 60.3 versus 53.8 years, *p* = 0.009). At last follow up, 152 of 288 patients in ongoing MRDneg-CR had undergone transplant during their treatment course, compared to 28 of 44 patients with REL-MRD and 41 of 100 with REL-MORPH.

The principal treatments given for REL-MRD were HCT (10 patients) and chemotherapy (17 patients: high intensity 6, intermediate intensity 3, and low intensity 8). A small number of patients who had already undergone HCT prior to REL-MRD underwent withdrawal of immunosuppression, with or without low intensity chemotherapy (2 patients and 4 patients respectively). 10 patients did not undergo any specific therapy for REL-MRD once identified. 1 patient’s treatment and response was unknown. Among patients who received treatment for their MRD, the median time to initiation of treatment for MRD was 24 days (interquartile range 10–36 days). Subsequently, another MRDneg-CR was observed in 12, death in 3, persistent REL-MRD in 10, and progression to REL-MORPH in 18. In this cohort, we did not identify any baseline factors associated with response to MRD therapy; time to MRD positivity was similar in all patients.

Seventy-nine patients died prior to the landmark date, with 40 in MRDneg-CR, 12 in REL-MRD, and 27 in REL-MORPH at the time of death. An additional 35 patients were censored as last contact was prior to the landmark date; all these patients were in ongoing MRDneg-CR. Among the subset of 318 patients alive at 1 year, 265 (83%) remained MRD negative, 23 (7%) were in REL-MRD, and 30 (9%) were in REL-MORPH (Table 1). No significant differences in age, gender, secondary disease, blasts <20% at diagnosis, or initial treatment intensity were identified across these groups of patients. Likewise, comparisons of patients who developed REL-MRD or REL-MORPH at a variety of timepoints following initial CR achievement (30–90, 90–150, and 150–210 day intervals were tested) showed no significant differences in patient characteristics, including age, gender, secondary disease, or underlying diagnosis.

**Table 1:** Patient characteristics by relapse category at 1 year.

Factor	Ongoing MRDneg-CR (*n* = 265)	REL-MRD (*n* = 23)	REL-MORPH (*n* = 30)	*P*-value
Age (years)	53.6 (42, 66)	60.8 (55, 68)	54.7 (45.2, 65)	0.1
Female	126 (48)	7 (30)	10 (33)	0.11
Secondary AML	93 (35)	13 (57)	9 (30)	0.098
Blasts <20% at diagnosis	49 (18)	5 (22)	7 (23)	0.69
ELN Risk at diagnosis				0.039
Favorable	101 (38)	2 (9)	10 (33)	
Intermediate	105 (40)	13 (57)	14 (47)	
Adverse	57 (22)	8 (35)	6 (20)	
Initial Treatment Intensity				0.93
High	172 (65)	14 (61)	20 (67)	
Intermediate	70 (26)	8 (35)	8 (27)	
Low	23 (9)	1 (4)	2 (7)	

Median (interquartile range) or *N* (%) reported.

*CR* complete remission, *MRD* measurable residual disease, *ELN* European LeukemiaNet 2017.

**Fig. 1:**
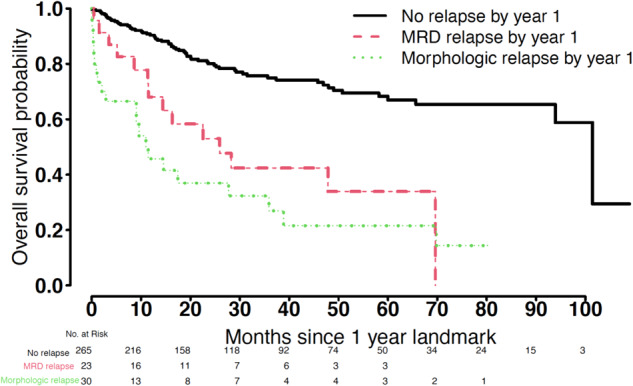
Overall survival using Kaplan-Meier estimates. A 1 year landmark analysis was performed to stratify outcomes for patients by relapse category, including no relapse, MRD relapse (REL-MRD), and morphologic relapse (REL-MORPH).

Among the 318 patients, MRDneg-CR was associated with longer OS than either REL-MRD or REL-MORPH (Fig. 1). In a multivariable regression model, MRDneg-CR remained associated with a lower risk of death than REL-MORPH [HR 0.17 (95% CI 0.1–0.29), *p* < 0.001]. Comparing OS suggests there is a decreased hazard for death among patients with REL-MRD compared to REL-MORPH, though the small sample size precludes a narrow enough confidence interval for more definitive conclusion [HR 0.52 (95% CI 0.25–1.06), *p* = 0.072].

In conclusion, following an initial MRDneg-CR in AML or high-grade myeloid neoplasm, development of either REL-MRD or REL-MORPH were both associated with decreased OS. In this cohort, notably, no significant differences in terms of OS were seen between patients who presented with MRD as opposed to morphologic relapse. There were no clear predictors identified for development of MRD or morphologic relapse based on baseline characteristics.

We observed significant heterogeneity among treatments received for MRD, which likely reflects lack of consensus regarding optimal treatment of MRD. Although no significant associations between type of MRD directed therapy and survival was identified, the sample size was small and limited ability to make comparisons.

Strengths of this study include the large number of patients in our database, widespread utilization of a consistent MFC method in the study population, and the use of landmark analysis to minimize survival bias. However, given that there is no standardized approach for the frequency of conducting MFC assessments in patients with MRDneg-CR, the monitoring patients received was variable. While our study relied on MFC for evaluation of MRD, other validated modalities of MRD assessment (next-generation targeted sequencing, cytogenetics, or fluorescence in situ hybridization) were not evaluated. Similarly, institutional variability in MFC utilization and protocols may limit the generalizability of this study.

Overall, this analysis adds to a growing body of work that demonstrates any detectable disease is associated with worse outcomes [10]. Whether REL-MRD has different clinical characteristics or necessitates different treatment strategies than persistent MRD level disease following induction therapy remains to be demonstrated. Importantly, the appropriate balance between treatment toxicity, efficacy of treatment, and benefit of treatment for REL-MRD is not yet known. Our findings demonstrate that even with treatment, patients with REL-MRD do poorly. In light of these findings, ongoing investigations and clinical investigations focused on MRD are warranted; however, evaluation of trials focused on MRD directed therapy are particularly challenging to interpret due to lack of validated surrogate endpoints.

## Data Availability

The datasets generated during the study are available from the corresponding author on reasonable request.
